# Serological evidence of natural exposure to rabies in rural populations in Gabon

**DOI:** 10.1371/journal.pntd.0012044

**Published:** 2024-11-14

**Authors:** Linda Bohou Kombila, Nadine N’dilimabaka, Julien Lannoy, Eric Elguero, Eric M. Leroy, Laurent Dacheux, Pierre Becquart

**Affiliations:** 1 Unité Émergence des Maladies Virales (UEMV), Département de Virologie, Centre Interdisciplinaire de Recherches Médicales de Franceville (CIRMF), Franceville, Gabon; 2 Ecole Doctorale Régionale d’Afrique Centrale en Infectiologie Tropicale (EDR), Université des Sciences et Techniques de Masuku (USTM), Franceville, Gabon; 3 Département de Biologie, Faculté des Sciences, Université des Sciences et Techniques de Masuku (USTM), Franceville, Gabon; 4 Institut Pasteur, Université Paris Cité, Unit Lyssavirus, Epidemiology and Neuropathology, WHO Collaborating Centre for Reference and Research on Rabies, Paris, France; 5 MIVEGEC, Institut de Recherche pour le Développement (IRD), Montpellier University, CNRS, Montpellier, France; 6 TRANSVIHMI, Université de Montpellier-IRD-Inserm 34394 Montpellier, France; The University of Kansas, UNITED STATES OF AMERICA

## Abstract

Rabies is one of the oldest known zoonotic diseases, with dogs being the main reservoir for 99% of the cases of human rabies. However, wild animals may also be rabies vectors. In most cases, contact with a rabid animal results in rabies without pre- or post-exposure prophylaxis, and the disease is nearly always fatal. Nevertheless, a few studies have documented cases of rabies-specific antibodies detection in people with no history of vaccination, suggesting that individuals can be in contact with the virus without developing fatal rabies. To further investigate this possibility of non-lethal human rabies exposure, we carried out a retrospective serological analysis, using both immunoassays (ELISA) and seroneutralization assays (RFFIT), on 430 sera collected between 2005 and 2008 from rural unvaccinated Gabonese populations in the Estuaire and Ogooué-Ivindo provinces. Eleven (11) samples (2.5%) were positive for rabies-specific antibodies using both techniques: 1 in Estuaire and 10 in Ogooué-Ivindo. One of three positive people from the Ogooué-Ivindo province, resampled in early 2023, was still positive for rabies-specific antibodies, suggesting that some degree of immunity can be maintained over many years. Our results also show a marginally significant higher prevalence among hunters. This study demonstrates that rabies circulates actively in Gabon and some unvaccinated individuals living in rural environments can be exposed to the virus and survive, with the development of a significant and specific humoral response that can persist for more than 15 years. This passive seroprevalence survey underlines the need to establish a national surveillance system of rabies in both humans and animals in urban and rural areas, and to enhance access to pre- and post-exposure prophylaxis.

## Author summary

Rabies is one of the oldest neglected tropical diseases transmitted to humans, generally by dogs, but wild animals can also act as vectors of the disease. In most cases, a risk-contact with an infected animal without proper prophylaxis results in rabies, and the disease is nearly always fatal. Nevertheless, a few studies have documented the detection of rabies-specific antibodies in people having no history of vaccination, suggesting that exposition to the virus is possible without developing fatal rabies. To further investigate this possibility, we carried out a retrospective serological analysis on 430 sera collected between 2005 and 2008 from rural Gabonese populations. We detected 11 positive samples (2.5%). Three of these positive people was resampled in early 2023 and one was still found positive for rabies-specific antibodies, suggesting that immunity can persist over many years. This study demonstrates that rabies is actively circulating in Gabon and that some unvaccinated individuals living in rural environments can be exposed to the virus and survive. Survival despite exposure to rabies could be associated to the development of a significant and specific humoral response that persists for more than 15 years.

## Introduction

Rabies is one of the most ancient infectious diseases known, with the first suggested descriptions dating from the 4^th^ century BC [1]. This disease is also one of the first to benefit from the development of a specific vaccine, developed by Louis Pasteur and Emile Roux [2]. Unfortunately, rabies still represents a major public health problem, particularly in endemic areas located mainly in low- and middle-income countries. An estimated 60,000 people die each year from this disease, mainly in Asia and in Africa [3], although this figure is probably underestimated and needs to be updated. Indeed, the number of countries presenting publicly available national strategic plans for rabies surveillance and control is still very limited, particularly in Africa.

The etiological agents of rabies are viruses belonging to the genus *Lyssavirus* within the family *Rhabdoviridae*. To date, a least 18 different lyssavirus species have been officially recognized, but this number is continuously increasing, with new isolates awaiting classification [4]. Nearly all have been discovered in bats, suggesting that the order Chiroptera represents the original reservoir of these viruses [5]. However, the species *Rabies lyssavirus*, represented by rabies virus (RABV), is widely distributed in other mammals, especially carnivores, and the domestic dog plays a crucial role in the epidemiology of human rabies [3]. Dogs are the main reservoir and vector of human rabies, transmitting the infection mainly by bites, or in some case through scratches or licking open wounds (as the virus does not pass through the skin intact) and mucous membranes.

Once in the body, the virus can remain at the point of inoculation for some time (incubation period), potentially multiplying in muscle cells or other local tissues. Then, the virus infects motor neuron axons and migrates in a centripetal fashion along the peripheral nervous system, ultimately reaching the central nervous system. Alternatively, it can enter the peripheral or central nervous system directly through decaying or deep wounds [6]. The prodrome stage occurs when the virus replicates in dorsal root ganglia before the syndromic stage, associated with the multiplication in the brain. Two main clinical forms are observed: paralytic and encephalitic forms [7]. Finally, the virus migrates centrifugally from the central nervous system, via the motor and sensory nerves, to innervated and non-nervous tissues. At this stage, the virus is exposed to the immune system and rabies-specific antibodies are detectable in the peripheral vascular system (around 10 days post-infection) [8,9].

In humans, like other mammals (excluding bats), once the first clinical signs appear, the outcome is considered nearly always fatal. Very rare cases of survival after confirmed rabies infection have been described, but most of the time, patients suffer significant neurological after-effects and have a short survival expectancy [10]. To date, there is no treatment for rabies once the symptoms have developed, and post-exposure prophylaxis must be implemented as soon as possible after exposure to rabies. Post-exposure prophylaxis consists in administering a series of rabies vaccines (inactivated virus), with or without serotherapy (human or equine rabies IgG, monoclonal antibodies), depending on the degree of exposure and on previous rabies vaccination (pre-exposure administration or previous post-exposure prophylaxis) [11].

Despite the dogma that rabies is nearly always fatal, the question of whether it is possible to be exposed or even be infected without developing the disease, remains open to debate. Not all cases of exposure to infected animals lead to the development of rabies, because the onset of the disease depends on various factors such as the severity and location of the bite, the viral load and the virus isolates/species. For example, it has been estimated that rabies mortality after untreated bites from rabid dogs varies from 38% to 57% [7]. Similarly, a limited number of studies have investigated the presence of rabies-specific antibodies in the sera of non-vaccinated and healthy individuals [12–23]. In some of these individuals, seropositivity against rabies has been detected, suggesting that they were naturally exposed to rabies, through professional or personal activities, without developing the disease [14–16,19,21,22]. Similar results have also been reported in various mammalian reservoirs and vectors, including domestic dogs and other wild carnivores [24]. However, depending on the study methodology, serological techniques and selected positivity thresholds, results can be difficult to interpret [24].

To extend these studies and further evaluate the possibility of non-lethal exposure to rabies in humans, we carried out a retrospective serological analysi on populations living in two provinces (Estuaire and Ogooué-Ivindo) in Gabon. Despite limited data on rabies circulation in this country due to the lack of rabies surveillance, rabies is considered endemic in the dog population, as in most other African countries [3,6,25]. Although largely underestimated, a few cases of human rabies have also been already described, demonstrating that this disease is present. In humans, previous cases reported to the WHO were diagnosed based on clinical symptoms alone. In 2004, a 3-year-old child with a history of travel to Gabon was diagnosed with furious rabies in France in 2003 [26]. More recently, two cases of human rabies in 2019 and 2020 in the capital Libreville were described and the first molecular diagnosis confirmed the presence of the virus in Gabon [27]. Because both patients had a history of dog bites a few weeks before the onset of symptoms, these animals were likely to be the source of human infection. In enzootic zones, it is also now established that populations are more exposed to rabies in rural areas [6]. However, it is precisely in these areas that access to rabies prevention and vaccination is extremely limited, if not non-existent [25]. In this epidemiological context and using combined serological techniques (ELISA and seroneutralization), we set out to evaluate the presence of rabies-specific antibodies in rural populations, and to demonstrate that some non-vaccinated individuals are seropositive for rabies. Our results show that, likely due to their hunting activities, rural populations may be exposed to rabies without developing an infection.

## Materials and methods

### Ethics statement

For retrospective samples (obtained during the period 2005–2008), the plasmas were collected as part of an initial clinical study focusing on the Ebola virus, and the related protocol was reviewed and approved by the Gabonese Ministry of Health (authorization nu00093/MSP/SG/SGAQM). Written consent was obtained from the Health Director of each region, the traditional chiefs of each village and each participant. At the time, study was described orally, and volunteers gave their signed informed consent to be enrolled in the study and for their blood samples to be used for future research studies (in link with virus infection).

For prospective samples (obtained in 2023), patients whose rabies serology was positive (by both ELISA and RFFIT tests) and who could be traced using the information available were contacted again, with the agreement of the traditional chief of the village concerned. After reminding them of the context of the initial study that collected their first blood sample (between 2005 and 2008), and of the current study on anti-rabies seroprevalence, they were asked to take a new blood sample in order to assess the persistence of these anti-rabies antibodies. On this occasion, the results of this serology were communicated to them, with a simple information given at the time, specifying that it was an indicator of past exposure to the infectious agent, and not associated with a disease. A new sample was taken only after oral consent had been obtained from the patients concerned.

### Study population and samples

**Fig 1:**
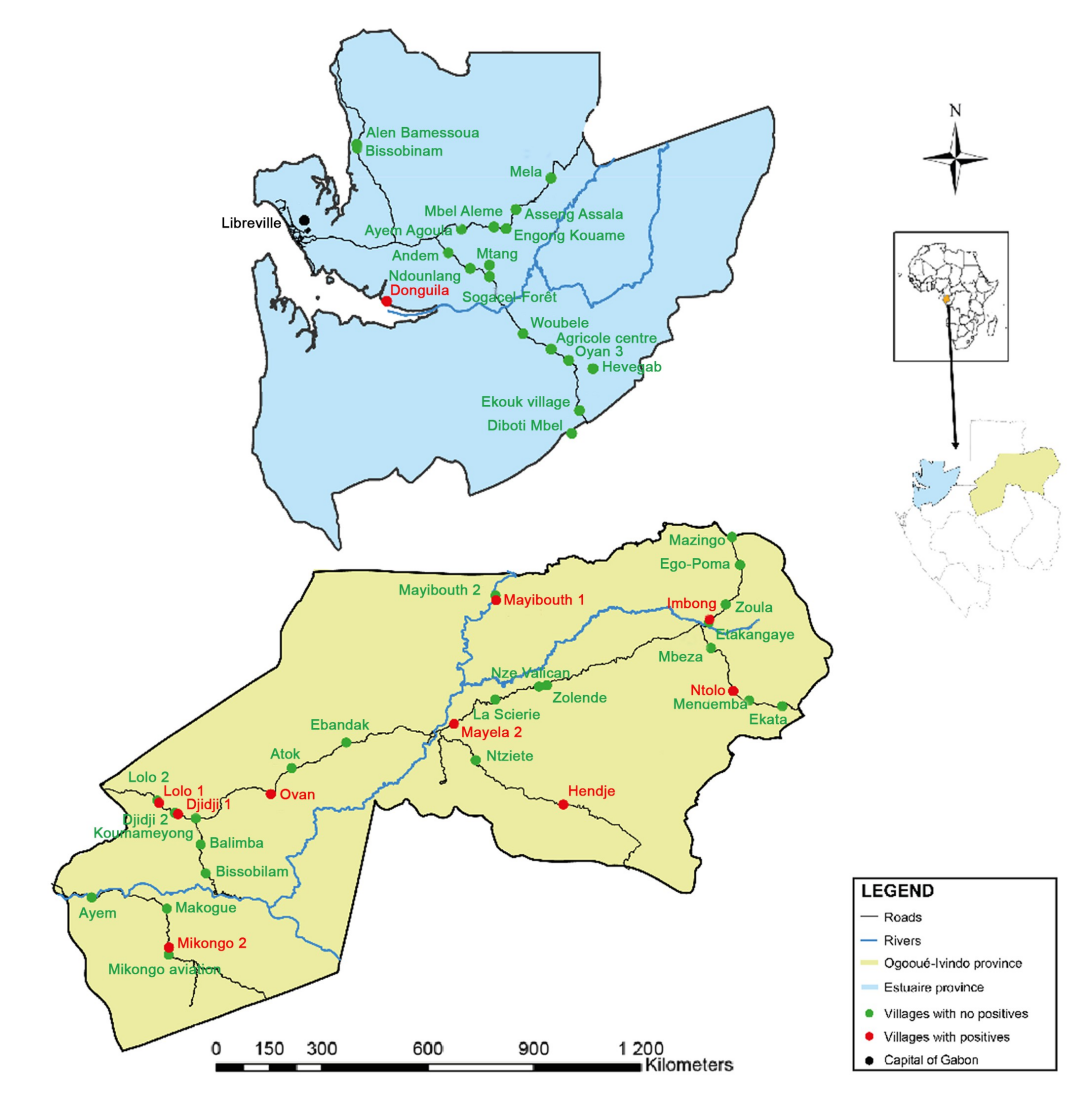
Location of the samples collected and analyzed in this study. The map was created using ArcMap 10.4.1 software. The source of the shapefile for the map background is https://gadm.org/data.html. The source of the routes and waterways is https://extract.bbbike.org/.

To conduct this study, we used retrospective plasma samples from our previous serosurvey conducted from June 2005 to September 2008 throughout Gabon [28]. Two geographical areas were selected for this study. The first area was the province of Estuaire, located in the west of Gabon on the Atlantic coast, which includes Libreville, the capital. This province is characterized by forests, mangroves and savannahs. In 2019 and 2020, two children died of rabies in the Estuaire province [27]. The second was the province of Ogooué-Ivindo, located in the north-east and made up mainly of tropical rainforests. The samples were collected from 100 individuals in 18 villages in Estuaire province and from 332 individuals in 31 villages in Ogooué-Ivindo province (Fig 1). None of the people included in the study had been vaccinated against rabies at the time of sampling. This survey initially focused on rural villages with fewer than 300 inhabitants, which is relevant for increasing the probability of identifying individuals naturally exposed to rabies. During this previous study, all permanent residents aged over 15 years were invited to participate and provide blood samples. Data such as sex, age, province, occupation, hunting activity and village environment were collected to study the risk factors associated with rabies exposure.

### Detection of specific anti-rabies IgG using ELISA and seroneutralization (RFFIT)

The detection of specific IgG against the glycoprotein of the rabies virus in plasma samples was conducted using a qualitative enzyme-linked immunosorbent assay (ELISA). The samples were decomplemented by heating for 30 minutes at 56°C, and ELISA was performed using the Platelia Rabies II Kit (BioRad, ref 3551180) according to the manufacturer’s recommendations, with the qualitative method [29]. All samples presenting a signal equal or above the positive control (0.5 equivalent units (EU)/mL) were considered positive. A confirmatory technique was performed using the rapid fluorescent focus inhibition test (RFFIT), as previously described [29–30]. This technique is a cell-based virus neutralization assay used to measure the level of protection against RABV in humans and animals. For this technique, performed in 96-well microplate format, a constant dose of previously titrated, cell culture adapted, RABV challenge virus strain (CVS) is incubated with serial dilution of the sera to be titrated. A reference serum of known titer is included in each test. After one hour of incubation at 37°C, BSR cells (a clone of BHK-21 cells) are added in each well. After 24 h incubation, the estimation of the percentage of infected cells for each dilution of the sera allows determination of the titer of the unknown sera by comparing with the reference serum. The detection and quantification limits of this technique, accredited and implemented according to the ISO 15189 standard, were 0.01 and 0.19 international units per mL (IU/mL), respectively. To avoid false positives and increase the stringency of this test for this study, samples with titer > 0.38 IU/mL (twice the limit of quantification) were considered positive. This test was performed on all ELISA-positive samples, as well as on a random subset of ELISA-negative samples from each province (27 and 40 samples from Ogooué-Ivindo and Estuaire, respectively), in order to validate the RFFIT positivity threshold.

### Statistical analysis and software

Forty ELISA-negative samples from Estuaire were randomly selected for seroneutralization using R 4.0.4. The associations at the individual level between the presence of antibodies and sex, age, main occupation (hunter, farmer, others) and hunting activity (professional or otherwise) were assessed using logistic regression. All calculations (odds ratio determination and Benjamini-Hochberg procedure) were performed with the R software and the results of the regression were analyzed for false discovery rate. Figures were produced using ArcMap 10.4.1 and Adobe illustrator 2020 (Fig 1) and GraphPad Prism 10.2.1 (Fig 3).

**Fig 2:**
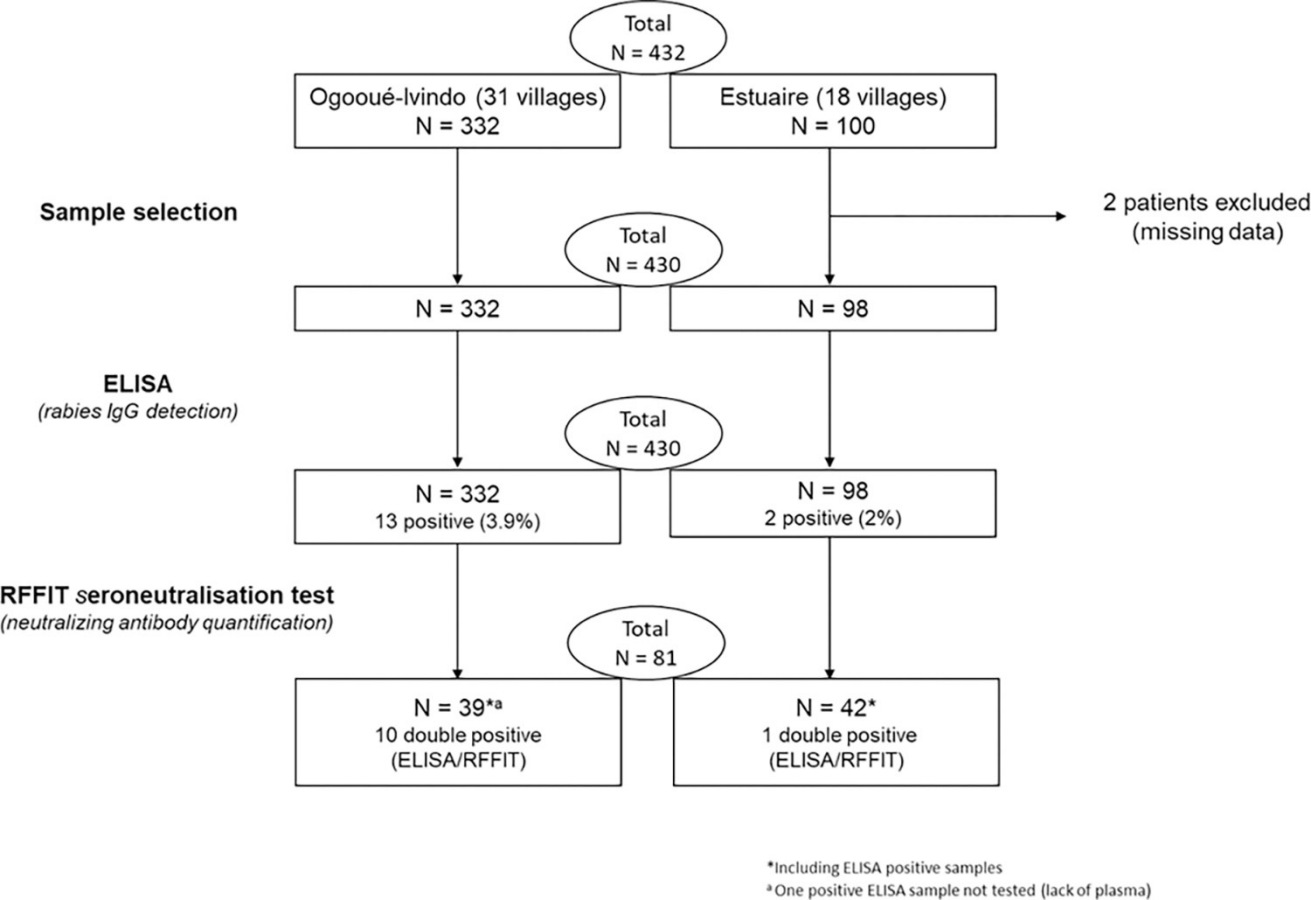
Flowchart of plasma sample selection and serological analysis.

## Results

### Description of the population

In Ogooué-Ivindo, a total of 332 plasma samples were collected (Figs 1 and 2 and Table 1). The study population according to sex included 180 (54.2%) men and 152 (45.8%) women. The ages ranged from 18 to 84 years old (mean age of 46.5). The most represented type of village environment was “forest” with 315 people (94.9%). Farmer was the most common occupation, with 204 individuals (61.5%) (Table 1). In Estuaire, a total of 100 plasma samples were collected, and 98 were analyzed (two samples were excluded due to missing data) (Fig 2). The study population according to sex included 50 (51.0%) men and 48 (49.0%) women. Their ages ranged from 18 to 65 years old (mean age of 46.1). The most represented type of village environment was “forest” with 86 people (87.7%). Farmer was also the most common occupation, with 69 (70.4%) individuals (Table 1).

**Table 1:** Descriptive statistical analyses of the study population according to geographical origin (provinces of Estuaire and Ogooué-Ivindo, Gabon) and serological results.

	Estuaire		Ogooué-Ivindo			
	Sample size	Positive samples^1^	Sample size	Positive samples^1^	OR [95% CI]^2^	p-value
**Sex**						
Men	50	0	180	7(3.9%)		
Women	48	1(2.1%)	152	3(2.0%)	2.0 [0.5–7.9]	0.31
**Age group**						
18–33	18	0	75	3(4.0%)	0.5 [0.1–2.9]	0.42
34–49	30	1(3.3%)	105	2(1.9%)	0.9 [0.2–4.1]	0.94
50–65	50	0	132	5(3.8%)	0.0 [0.0–Inf]	0.99
≥66	0	0	20	0		
**Main occupation**						
Farmer	69	1(1.4%)	204	4(2.0%)	0.5 [0.1–2.8]	0.42
Hunter	3	0	77	4(5.2%)	1.2 [0.2–7.6]	0.74
Other	26	0	51	2(3.9%)		
**Hunting activity**						
None	61	1(1.6%)	221	3(1.4%)		
Hunting	24	0	111	7(6.3%)	4.9 [1.2–19.5]	0.023*
N/A	13	0	0	0		
**Village environment**						
Forest	86	0	315	10(3.2%)	-	
Lagoon	12	1(8.3%)	0	0	-	
Savannah	0	0	17	0	-	
**Total positive samples**		**1/98 (1.0%)**		**10/332 (3.0%)**		

^1^Samples positive in both ELISA and seroneutralization tests.

^2^Odds ratio (95% confidence interval).

### Rabies seroprevalence

#### Rabies IgG detection using ELISA

All the selected samples (n = 430) were tested using ELISA based on the qualitative method of the Platelia Rabies II kit to detect IgG specific to the glycoprotein of the rabies virus. A total of 15 samples (3.5%) were considered positive (titer ≥ 0.5 EU/mL) (Table 2).

**Table 2:** Results of seroneutralization assay (RFFIT) on positive ELISA samples.

Number ID	Hunting activity	Province	Location (village)	ELISA result	RFFIT titer (IU/mL)	RFFIT result
78–58	No hunting	Estuaire	Donguila	** + **	0.95	** + **
78–65	No hunting	Estuaire	Donguila	** + **	0.26	-
1030–01	Hunting	Ogooué-Ivindo	Ovan	** + **	0.95	** + **
1003–08	No hunting	Ogooué-Ivindo	Lolo 1	** + **	1.05	** + **
998–31	No hunting	Ogooué-Ivindo	Djidji	** + **	1.05	** + **
1064–07	Hunting	Ogooué-Ivindo	Ntolo	** + **	1.05	** + **
1503–12	Hunting	Ogooué-Ivindo	Mayibouth 1	** + **	1.05	** + **
1469–04	Hunting	Ogooué-Ivindo	Hendje	** + **	1.05	** + **
1469–12	Hunting	Ogooué-Ivindo	Hendje	** + **	1.05	** + **
1489–01	Hunting	Ogooué-Ivindo	Mayela	** + **	1.05	** + **
964–04	No hunting	Ogooué-Ivindo	Mikongo 2	** + **	3.14	** + **
1068–09	Hunting	Ogooué-Ivindo	Imbong	** + **	1.05	** + **
1071–10	No hunting	Ogooué-Ivindo	Mazingo	** + **	0.22	-
1469–19	Hunting	Ogooué-Ivindo	Hendje	** + **	N/A	N/A
1050–18	Hunting	Ogooué-Ivindo	Ekata	** + **	0.38	-
20–139*	Hunting	Estuaire	Ay. Agoula	-	0.67	** + **
1021–05*	Hunting	Ogooué-Ivindo	Atok	-	0.95	** + **
964–10*	No hunting	Ogooué-Ivindo	Mikongo 2	-	1.05	** + **

*ELISA-negative samples found positive with RFFIT (n = 3) among the random subset of ELISA-negative samples (n = 67)

#### Confirmation using the RFFIT seroneutralization assay

**Fig 3:**
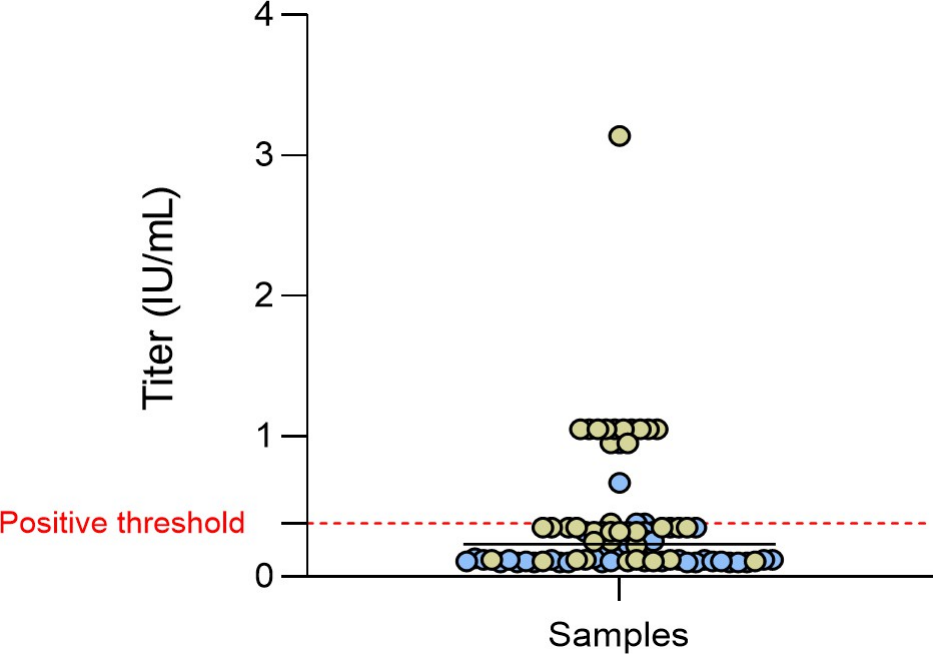
Distribution of the seroneutralization titers for each plasma sample tested using RFFIT. Green and blue circles correspond to samples from Ogooué-Ivindo and Estuaire provinces, respectively. The red dashed line represents the positive cut-off titer value (> 0.38). Black bar indicates the mean value. Neutralization titers are indicated in international units per mL (IU/mL).

Of the 15 ELISA-positive samples, 14 were screened for confirmation using the seroneutralization assay (RFFIT) (12 from Ogooué-Ivindo and 2 from Estuaire). The remaining ELISA-positive sample (#1469–19) could not be tested due to insufficient quantity of plasma (Table 2). Among them, 11 ELISA-positive samples (78.6%) were confirmed with the RFFIT (cut-off value > 0.38 IU/mL) (Table 2 and Figs 2 and 3). The seroneutralization titer ranged from 0.95 to 3.14 IU/mL (median value 1.05 IU/mL).

One was from Estuaire, whereas the ten others were from Ogooué-Ivindo (Fig 2 and Tables 1 and 2). The majority of the positive individuals was men (7/11, 63.3%), and lived in forest environment (10/11, 90.9%). The main activities of the positive individuals were farming (n = 5), hunting (n = 4), sawmilling (n = 1), and one was unemployed. Seven of the 11 positive people practiced hunting. When referring to the number of samples initially tested only by ELISA, only samples that tested positive with ELISA and seroneutralization were considered positive. Therefore, the overall positivity rate was 1.0% (1/98) and 3.0% (10/332) for Estuaire and Ogooué-Ivindo, respectively.

Among the random subset of ELISA-negative samples tested to validate the positivity cut-off (27 and 40 samples from Ogooué-Ivindo and Estuaire, respectively), three ELISA-negative samples (3/69; 4.5%) (#20–139, #964–10 and #1021–05) were found positive with the RFFIT, with titers ranging from 0.67 to 1.05 IU/mL (Table 2).

To estimate the persistence of the immune response against rabies, we were able to go back and retest some individuals who had positive ELISA results. Among the 13 ELISA-positive from Ogooué-Ivindo, 3 individuals (#1068–09, #1469–04 and #1469–19) could be resampled in March 2023, and plasma was collected after obtaining their oral consent. None of these three individuals has been vaccinated between the two-sampling dates. Plasmas were tested by both ELISA and RFFIT, and only one (#1469–04), a farmer, tested positive by ELISA, subsequently confirmed by the seroneutralization assay (0.95 IU/mL). The two other samples were negative using both techniques (S1 Fig).

### Risk factor analysis

Given the small number of positive samples from Estuaire, statistical analyses were carried out only on Ogooué-Ivindo samples (Table 1). The statistical treatment of all samples (from both provinces) is indicated in Supplementary Information (S1 Table). In Ogooué-Ivindo, only hunting activity was associated with the presence of antibodies (Table 1). The odds ratio associated with hunting calculated from the logistic regression was 4.9, with a 95% confidence interval [1.2–19.5] and a p-value equal to 0.023. After correction for false discovery rate using the Benjamini-Hochberg procedure, the association was no longer statistically significant (effective p-value = 0.092). Statistical analysis carried out on both provinces gave similar results (odds ratio for hunting: 3.8 [1.1–13.3], p-value = 0.035) (S1 Table).

## Discussion

Rabies is an infection considered almost 100% lethal once clinical signs appear. However, little information is available on the possibility of exposure to the viral etiological agent (the main one being rabies virus) without subsequent infection leading to the development of disease. To shed light on this aspect of rabies epidemiology, we assessed the existence of natural exposure to non-lethal rabies in unvaccinated populations living in enzootic areas. To reach this goal, we evaluated seropositivity to rabies in retrospective plasma samples collected from rural populations living in two different provinces of Gabon [28]. The first province, Estuaire, features forests, mangroves and savannas, is located in the northwest of the country and includes Libreville; the second, Ogooué-Ivindo province, is located in the northeast and is mainly made up of tropical rainforests.

Our results demonstrate that some unvaccinated individuals can be naturally exposed to rabies and survive, while developing a humoral response to rabies virus that can reach levels comparable to those considered protective by the WHO (≥ 0.5 IU/mL or EU/mL) [11]. To date, only a limited number of studies have detected the presence of seropositivity against rabies in non-vaccinated individuals, suggesting that these individuals had been naturally exposed to rabies without developing the disease [14–16,19,21,22]. This natural exposure to rabies has also been observed in various mammalian reservoirs and vectors, including domestic dogs [24]. However, the results obtained by these studies are difficult to compare, due to the differences between the techniques used and/or the choice of positivity thresholds selected. [24]. Various techniques were used in those previous studies, such as the mouse inoculation test (MIT) [12,13], the complement fixing antibodies test (CF) [18], the indirect fluorescent antibody test (IFA) [21] or seroneutralization assays with the RFFIT [14–17,19–23]. Even for the latter technique, some results were expressed in terms of dilution factor instead of IU/mL [16] or used a lyssavirus other than rabies virus (CVS strain) [23].

Finally, a very small number of studies have been able to detect neutralizing antibody titers in a few unvaccinated patients, in a way that makes comparison possible. For example, during an outbreak of human rabies in two rural communities in the Amazon jungle in Peru, the median titer of seven positive healthy and unvaccinated people (among 47) was 0.18 IU/mL, ranging from 0.14 to 0.66 IU/mL [17]. In another study, only 1 unvaccinated individual out of 21 exhibited a 2.3 UI/mL titer in a trapper community from Alaska, subsequently confirmed by another lab [19]. Investigation of rabies virus exposure in two communities living in the Peruvian Amazon demonstrated the presence of neutralizing antibodies in six unvaccinated individuals, with median 0.5 IU/mL (range 0.1–2.8 IU/mL) [21]. In another study, three individuals exposed to dog meat in Nigeria (butcher or dog meat consumers) had neutralizing antibody titers ranging from 0.65 to 0.7 IU/mL [20], without any antecedent of vaccination. Lastly, a study in Nunavik identified two unvaccinated individuals in a subset (n = 196) of the Canadian Inuit population with titers of 0.51 and 0.71 IU/mL [22]. In comparison, our study reports one of the highest numbers of cases of unvaccinated people seropositive against rabies (n = 11) with neutralizing antibodies titers reaching up to 3.14 IU/mL.

To minimize the difficulties in interpreting this type of result, and ultimately to limit the risk of false positives, we used a combination of two complementary and successive serological techniques—ELISA and RFFIT—in our study. Both techniques have been validated by the WHO for the determination of rabies antibodies after pre- or post-exposure prophylaxis or in cases of rabies infection in humans. Combining these tests also allowed us to measure different characteristics of the rabies humoral response. ELISA detects the presence of specific anti-rabies IgG, whereas the RFFIT test measures the neutralizing activity of antibodies, whatever the class of immunoglobulin considered. In addition, high positive thresholds for each of these techniques were selected. For instance, only plasma samples ≥ 0.5 EU/mL were considered positive (qualitative method of the Platelia Rabies II Kit) for ELISA, whereas this technique has a limit of detection of 0.05 UE/mL and a limit of quantification of 0.125 EU/mL, with ± 0.06 EU/mL uncertainty [29]. Similarly, RFFIT has a limit of detection of 0.05 IU/mL and a limit of quantification of 0.19 IU/mL, with 22% uncertainty, but we set the positive threshold at twice the detection limit (0.38 IU/mL). Therefore, this high level of stringency probably led to an underestimation of seroprevalence. In particular, given the high qualitative ELISA positivity threshold (≥ 0.5 EU/mL), it is not surprising that we detected seroneutralization-positive samples in our subset of ELISA-negative samples (Table 2). Similarly, discrepancies between the ELISA and RFFIT techniques, even if they are limited, do exist, notably because of the difference in methodological approach: the ELISA technique detects anti-rabies IgG whereas the RFFIT technique detects the neutralizing activity of plasma, whatever the immunoglobulin class [29]. The two complementary serological techniques used in this study are based on the use of the RABV CVS strain, limiting the risk of cross-reactivity with another lyssavirus from either of these techniques. However, it cannot be totally ruled out that one of them may also react with a new lyssavirus very similar to RABV. This is particularly true given the fact it that some patients vaccinated with RABV vaccine strain can develop cross-neutralizing antibodies to other phylogroup 1 lyssaviruses [31]. The techniques used in this study cannot assess this exposure, which may contribute to further underestimation of the seroprevalence of this disease, particularly among people regularly exposed to wildlife, such as hunters. The relatively low population size may represent another weakness of this study, because it is not possible to estimate an accurate seroprevalence rate in the two geographical areas of interest. Despite these limitations, our results highlight that some people may have naturally acquired immunity to the rabies virus in these regions.

The existence of patients who have been naturally exposed to rabies and survived, and who develop an efficient anti-rabies humoral response, remains intriguing. The detection of humoral immunity against rabies in unvaccinated and healthy individuals may be the result of an alternative course of rabies exposure [24]. According to Fekadu [32], four scenarios can lead to rabies antibody detection in healthy individuals: subclinical infection, recovery from clinical infection, carrier state, and extended latent period. From all these potential alternative courses, subclinical infection remains the most likely, both in humans and in animals, because it corresponds to a minimal infection sufficient to initiate the development of a specific immune response and to clear the virus before the onset of recognizable clinical symptoms. A fifth possibility may be repeated immunogenic exposure to small amounts of viral antigens, leading to the development of specific humoral immunity, as in rabies vaccination. In humans, these last two alternatives remain closely linked to hunting and farming activities that lead to this type of rabies exposure.

Both of these latter hypotheses are compatible in our study. Positive individuals may have been exposed to rabies virus *via* saliva, generally through bites, scratches or direct contact of mucous membranes with domestic or wild animals, through their activities and without infection or followed by a short infection. Our results show a marginally significant higher prevalence among hunters, who are more likely to be exposed to the rabies, particularly to wild animals of genera such as *Cephalophus* or *Philantomba* (antelopes), *Cercopithecus* (monkeys), *Potamochoerus* (bushpigs), *Genetta* (genets) and bats. Handling these animals may expose the person who caught or prepared them to the virus. Thus, it is possible that rabies antigen stimulation or aborted infection occur when villagers handle animals that have been contaminated by animal saliva and may contain infectious virus particles, inactivated virus particles or simple viral antigens. In addition, bats, which are reservoirs for the virus, circulate at night near homes, and are also eaten by some rural populations in Gabon [33] who would likewise be exposed to the virus. Similarly, individuals with rabies neutralizing antibodies described in previous studies carried out activities at risk of rabies exposure, such as trapping or hunting [19,22], exposure to dog meat [20] or living in remote areas populated by vampire bats in South America [17,21]. Moreover, in our study, three seropositive individuals were found and recontacted in early 2023, almost 15 years later after the first sampling. Among them, anti-rabies IgG were present in one person based on ELISA and confirmed with the seroneutralization assay. Interestingly, this individual, a farmer, has no memory of ever being bitten by a dog or another animal. Although we cannot rule out the hypothesis of minimal or undetected exposure during this period, this result would show that developed immunity can be maintained over many years.

Our results also demonstrate that rabies is present in the rural forest environment in Gabon and may represent a risk to the population, especially people who can be exposed due to their activities such as hunters. However, little information is available on the circulation of rabies virus in dogs, or other lyssaviruses in wildlife, and needs to be further investigated, in particular through the introduction or extension of post-mortem diagnosis of animal rabies on brain biopsy. This information is critical for understanding the transmission of the disease within these populations. In Gabon, vaccination campaigns have already taken place, organized by the Ministry of Livestock, the Ministry of Health and FAO (Food and Agriculture Organization). However, these campaigns are not recurrent and do not cover the entire territory. There is also very little rabies diagnostic capacity (notably the Centre international de recherches mùdicales de Franceville (CIRMF, Franceville, Gabon)), which has a further negative impact on rabies surveillance in Gabon. In addition, access to pre- or post-exposure prophylaxis is very difficult in remote rural areas due to the lack of health centers.

## Conclusion

In this study, we demonstrated that some unvaccinated individuals in the rural population in Gabon can be exposed to rabies and survive, and also develop a significant and specific humoral response. Despite the limitations of this study, such as modest sample size and the likely underestimation of the rabies seroprevalence, our results provide evidence for alternative modes of rabies infection in humans, and in particular the potential existence of subclinical infections that can lead to viral clearance with no apparent clinical signs or associated lethality. These results, combined with the few studies describing cases of human or animal rabies, also suggest that rabies is actively circulating in Gabon [27], and stress the need to enhance the surveillance of this disease both in humans and animals, as well as to raise public awareness and to improve access to post-exposure prophylaxis. This last aspect is particularly relevant and achievable with the recent inclusion of human rabies vaccines in Gavi’s vaccine investment strategy, the Vaccine Alliance for 2021–2025 [34]. All these actions are the key to achieving the goal of eradicating dog-related human rabies by 2030 [35].

## Supporting information

S1 Table Descriptive statistical analyses of the combined study population (from provinces of Estuaire and Ogooué-Ivindo, Gabon) according to serological results. (DOCX)

S1 Fig Distribution of the seroneutralization titers obtained by RFFIT for prospective plasma samples (collected in 2023) and corresponding retrospective plasma samples (when available, collected during the period 2005–2008). Each color corresponds to plasma samples from a same individual (n = 3). The red dashed line represents the positive cut-off titer value (> 0.38). Neutralization titers are indicated in international units per mL (IU/mL).
(TIFF)
