# Supplementary material for: Serological evidence of natural exposure to rabies in rural populations in Gabon
Source: PLoS Negl Trop Dis. 2024 Nov 14;18(11):e0012044. doi: 10.1371/journal.pntd.0012044 (PMC11594427; doi:10.1371/journal.pntd.0012044)
Supplement: S1 Table — (DOCX) [file pntd.0012044.s001.docx]

Table S1. Descriptive statistical analyses of the combined study population (from provinces of Estuaire and Ogooué-Ivindo, Gabon) according to serological results.

|  | **Sample size** | **Positive samples^1^** | **OR (95% CI)^2^** | **P-value** |  |
| --- | --- | --- | --- | --- | --- |
| **Province** |  |  |  |  |  |
| Estuaire | 98 | 1 |  |  |  |
| Ogooué-Ivindo | 332 | 10 | 3.0 [0.4 - 23.9] | 0.30 |  |
| **Sex** |  |  |  |  |  |
| Male | 230 | 7 (3%) | 1.5 [0.4 - 5.4] | 0.49 |  |
| Female | 200 | 4 (2%) |  |  |  |
| **Age groups** |  |  |  |  |  |
| 18 - 33 | 93 | 3 (3.2%) |  |  |  |
| 34 - 49 | 135 | 3 (2.2%) | 0.7 [0.1 - 3.5] | 0.65 |  |
| 50 - 65 | 182 | 5 (2.7%) | 0.8 [0.0 - 3.6] | 0.82 |  |
| 66+ | 20 | 0 (0%) | 0.0 [0.0 - Inf] | 0.99 |  |
| **Main activity** |  |  |  |  |  |
| Farmer | 273 | 5 (1.8%) | 0.7 [0.1 - 3.7] | 0.68 |  |
| Hunter | 80 | 4 (5.0%) | 2.0 [0.3 - 11.1] | 0.44 |  |
| Others | 77 | 2 (2.6%) |  |  |  |
| **Hunting activity** |  |  |  |  |  |
| No hunting | 282 | 4 (1.4%) |  |  |  |
| Hunting | 135 | 7 (5.1%) | 3.8 [1.1 - 13.3] | **0.035 *** |  |
| N/A | 13 |  |  |  |  |
| **Ecosystem** |  |  |  |  |  |
| Lagoon | 12 | 1 (8.3%) |  |  |  |
| Forest | 401 | 10 (2.5%) | 0.3 [0.0 - 2.4] | 0.25 |  |
| Savannah | 17 | 0 (0%) | - | - |  |
| **Total** | **430** | **11 (2.6%)** |  |  |  |
|  |  |  |  |  |  |
| ^1^Samples positive in both ELISA and seroneutralisation tests. | | | |  |  |
| ^2^Odds ratio (95% confidence interval). | | |  |  |  |
|  |  |  |  |  |  |
