# Supplementary figures and images for: Serological evidence of natural exposure to rabies in rural populations in Gabon
Source: PLoS Negl Trop Dis. 2024 Nov 14;18(11):e0012044. doi: 10.1371/journal.pntd.0012044 (PMC11594427; doi:10.1371/journal.pntd.0012044)

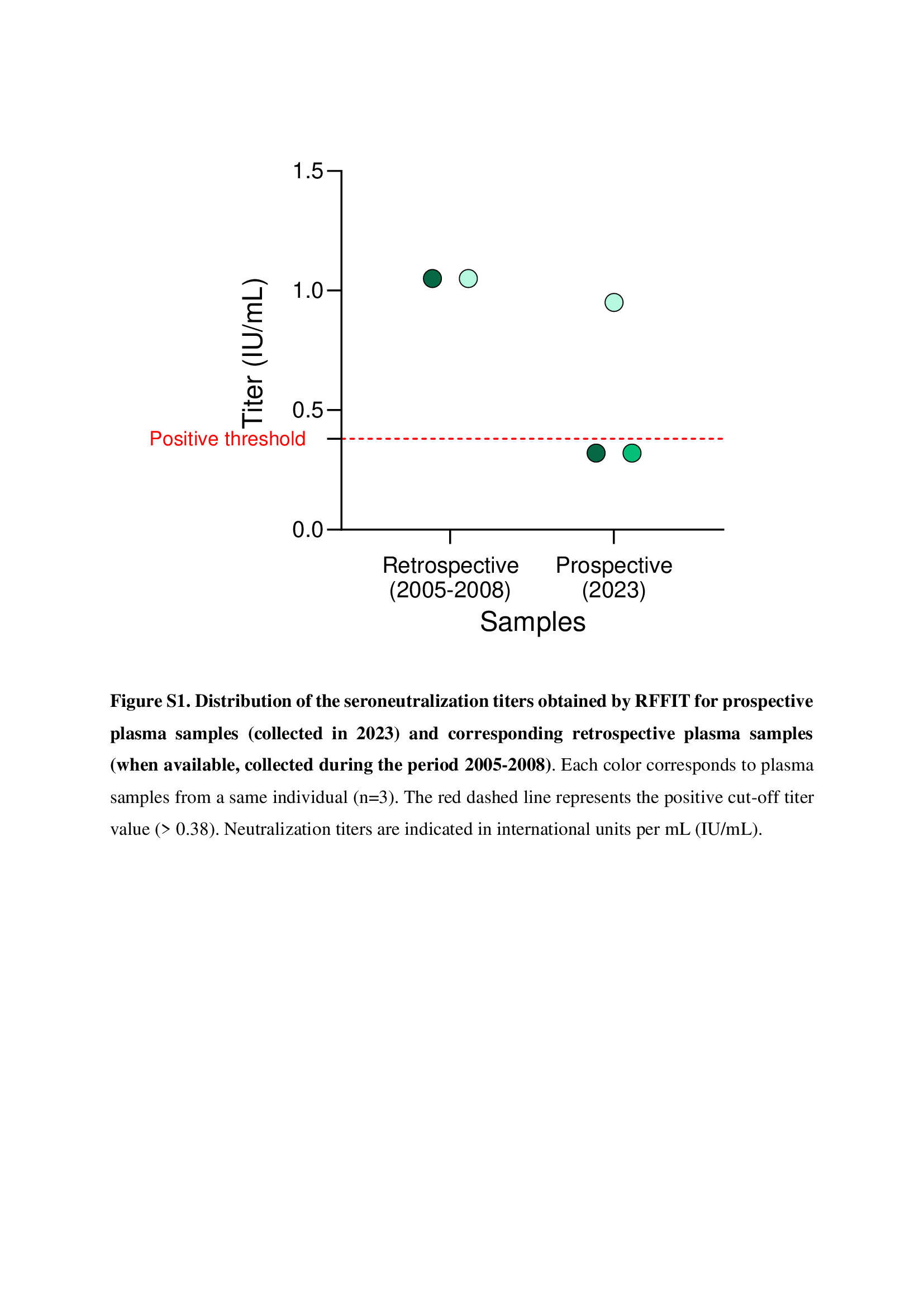

Supplement: S1 Fig — Each color corresponds to plasma samples from a same individual (n = 3). The red dashed line represents the positive cut-off titer value (> 0.38). Neutralization titers are indicated in international units per mL (IU/mL). (TIFF) [file pntd.0012044.s002.tiff]
